# The Acute Effects of Breakfast Drinks with Varying Protein and Energy Contents on Appetite and Free-Living Energy Intake in UK Older Adults

**DOI:** 10.3390/geriatrics7010016

**Published:** 2022-01-30

**Authors:** Daniel R. Crabtree, Adrian Holliday, William Buosi, Claire L. Fyfe, Graham W. Horgan, Alexandra M. Johnstone, on behalf of the Full4Health-study Group

**Affiliations:** 1 Division of Biomedical Sciences, University of the Highlands and Islands, Old Perth Road, Inverness IV2 3JH, UK; 2 Human Nutrition Research Centre, Population Health Sciences Institute, Newcastle University, Newcastle upon Tyne NE4 5PL, UK; adrian.holliday@newcastle.ac.uk; 3 The Rowett Institute, University of Aberdeen, Foresterhill Road, Aberdeen AB25 2ZD, UK; williambuosi@hotmail.fr (W.B.); c.fyfe@abdn.ac.uk (C.L.F.); alex.johnstone@abdn.ac.uk (A.M.J.); 4 Biomathematics and Statistics Scotland, Foresterhill Road, Aberdeen AB25 2ZD, UK; g.horgan@abdn.ac.uk

**Keywords:** ageing, protein, appetite, energy intake, BMI

## Abstract

Proposed strategies for preventing protein deficiencies in older patients include increasing protein intake at breakfast. However, protein is highly satiating and the effects of very high protein intakes at breakfast on subsequent appetite and free-living energy intake (EI) in older adults are unclear. This study compared the acute effects of two breakfast drinks varying in protein and energy contents on appetite and free-living EI in healthy older adults using a randomized 2 × 2 crossover design. Participants (*n* = 48 (20 men, 28 women); mean ± SD age: 69 ± 3 years; BMI: 22.2 ± 2.0 kg·m^−2^; fat-free mass: 45.5 ± 8.0 kg) consumed two drinks for breakfast (high-protein (30.4 ± 5.3 g), low-energy (211.2 ± 37.1 kcal) content (HPLE) and very high-protein (61.8 ± 9.9 g), fed to energy requirements (428.0 ± 68.9 kcal) (VHPER)) one week apart. Appetite perceptions were assessed for 3 h post-drink and free-living EI was measured for the remainder of the day. Appetite was lower in VHPER than HPLE from 30 min onwards (*p* < 0.01). Free-living energy and protein intake did not differ between conditions (*p* = 0.814). However, 24 h EI (breakfast drink intake + free-living intake) was greater in VHPER than HPLE (1937 ± 568 kcal vs. 1705 ± 490 kcal; *p* = 0.001), as was 24 h protein intake (123.0 ± 26.0 g vs. 88.6 ± 20.9 g; *p* < 0.001). Consuming a very high-protein breakfast drink acutely suppressed appetite more than a low-energy, high-protein drink in older adults, though free-living EI was unaffected. The long-term effects of adopting such a breakfast strategy in older adults at high risk of energy and protein malnutrition warrants exploration.

## 1. Introduction

Advancing age alters food reward signals [1], reduces food craving behavior [2], and suppresses appetite and energy intake (EI) [3], all of which contribute to a condition termed the “anorexia of ageing” [4]. Compared with younger adults, older adults are reported to consume approximately 30% less energy per day [5]. Dietary diversity (defined as the number of different foods or food groups consumed over a given reference period [6]) is also attenuated with ageing, with lower consumption of protein reported in older populations [7]. Inadequate regulation of food and protein intake increases the risk of developing conditions such as sarcopenia and osteoporosis [8]. Therefore, protein-energy homeostasis is considered a fundamental dietary-related determinant of healthy aging.

The World Health Organization recommends that adults consume 0.8 g protein·kg body mass^−1^·day^−1^ [9], while in the UK, the Reference Nutrient Intake is 0.75 g protein·kg body mass^−1^·day^−1^ [10]. However, these guidelines do not consider age. Dietary protein requirements increase with age, attributed partly to an increase in anabolic resistance to muscle protein synthesis (MPS), which accelerates loss of skeletal muscle mass and function [11,12]. More recent recommendations regarding optimal protein requirements for older adults suggest intakes of at least 1–1.2 g protein·kg body mass^−1^·day^−1^ for healthy older adults, 1.2–1.5 g protein·kg body mass^−1^·day^−1^ for older adults with acute or chronic disease, and as much as 2 g protein·kg body mass^−1^·day^−1^ for malnourished older adults [13,14]. Muscle protein synthesis research supports alternative daily recommendations for older adults, suggesting that a dietary protein intake of 20–30 g per meal of high-quality protein would sufficiently stimulate MPS in older adults [15,16], though further increasing protein intake may optimize muscle anabolism acutely [17].

Recently published data from the PRevention Of Malnutrition In Senior Subjects in the EU (PROMISS) project reported that, in a sample comprising of 8107 community-dwelling older adults, the prevalence of protein intake below 0.8 g·kg adjusted body weight (aBW)^−1^·day^−1^ was substantial (14–30%) and increased to 65–76% according to a cut-off value of 1.2 g·kg aBW^−1^·day^−1^ [18]. Furthermore, insufficient protein consumption is frequently reported in institutionalized older adults [19,20], with Leistra et al. [21] observing that of 610 undernourished hospitalized older adults, only 28% achieved protein requirements. Proposed strategies for preventing protein deficiencies in older adults include increasing protein intake at breakfast [19,20,22]. However, protein is highly satiating and the dose-dependent satiating effects of protein are well established [23,24]. Increasing protein intake at breakfast in an older population could support body mass and skeletal muscle mass retention and gain, though the satiety-inducing effects of protein may be detrimental to subsequent energy and protein intake, thereby adversely affecting muscle anabolism.

Interestingly, evidence suggests that older adults exhibit a blunted satiety response to protein consumption compared with younger adults [25,26,27]. Giezenaar et al. [26] compared the acute effects of two preload drinks differing in whey protein composition (30 g vs. 70 g) on EI at a buffet meal and appetite in healthy older and younger men. The authors found that the protein preloads suppressed buffet EI in both cohorts compared with the control preload (0 g protein), though suppression was less in older (1%) than younger (15%) men. Consequently, cumulative EI (preload drink + buffet EI) was greater in older (18%) compared to younger (1%) men. However, energy intake was not measured beyond this buffet meal, precluding any assessment of compensatory changes in eating behavior later in the day, and of the effects of large breakfast protein intakes on whole-day protein and energy intake. Furthermore, in a cross-sectional study, Verreijen et al. [28] reported that a higher self-reported protein consumption (≥30 g) at breakfast did not compromise protein intake at later eating occasions in community-dwelling older adults and was indeed associated with higher daily protein intake. However, protein intakes reported were modest, with only 1% of the 498 participants exceeding the recommended 0.4 g protein·kg body mass^−1^ per serving at breakfast. The acute and later-day appetite and food intake responses to very large protein intakes at breakfast in older adults, therefore, remain unknown.

The primary aim of this study was to determine the acute effects of two breakfast drinks, varying in protein and energy content (providing high and very high protein intakes), on appetite perceptions and free-living, whole-day protein and energy intake in community-dwelling older adults. Furthermore, factors influencing appetitive and dietary responses to breakfast protein and energy intake, including BMI, fat-free mass (FFM) and gender, were also investigated. To date, the influence of such factors in older adults is yet to be fully elucidated.

## 2. Materials and Methods

### 2.1. Recruitment and Participants

Men and women aged 65–75 years, with a BMI of <30 kg·m^−2^ were recruited by public advertisement using radio, newspapers and social media. Participants were also recruited from primary care, with support from the NHS Research Scotland Primary Care Network. The study exclusion criteria included: heavy smoking (more than 10/day); medication known to influence appetite; self-report fever/systemic infection; participation in medical or surgical weight loss program within 1 month of selection; current major depressive disorder; history of drug or alcohol misuse; >6 h of vigorous physical activity/week. This study was conducted according to the guidelines laid down in the Declaration of Helsinki [29]. Ethical approval was granted by the National Health Service North of Scotland Research Ethics Service (reference: 12/NS/0007; protocol number: 2/091/11). All participants provided written informed consent before entering the study. This trial was registered at www.clinicaltrial.gov (NCT01597024) (accessed on 7 December 2021).

Of the 50 participants enrolled on this study, 48 were included for analysis (See Figure S1 for details of recruitment, enrolment, and attrition). For the purpose of secondary analysis, participants were grouped for BMI as low BMI (<23.0 kg·m^−2^) and healthy BMI (≥23.0–29.9 kg·m^−2^). Such cut-offs were informed by a meta-analysis of 32 studies exploring BMI and mortality risk in older adults, which demonstrated that mortality risk increases when BMI was <23 kg·m^−2^ and above 33 kg·m^−2^, compared with “healthy-weight” BMI range of 23–33 kg·m^−2^ [30]. Participants were grouped for FFM as low FFM (<53.2 kg for men, <38.4 kg for women) and healthy FFM (≥53.2 kg for men, ≥38.4 kg for women), using gender-specific cut-off values of the 25th centile for FFM in a sample of 162 men and 183 women aged 65–74 years [31]. Participant characteristics for the entire sample and for each subgroup of gender, BMI and FFM are shown in Table 1. When grouped by gender, men (*n* = 20) and women (*n* = 28) did not differ by age or BMI, but FFM was greater in men than women (*p* < 0.001). When grouped by BMI, BMI was significantly lower in the low BMI group compared with the high BMI group (21.1 ± 1.4 kg·m^−2^ vs. 24.4 ± 1.0 kg·m^−2^; *p* > 0.001) but groups did not differ by age or FFM. Body composition data was not obtained from five participants; therefore, FFM data was analyzed for 43 participants. FFM, was significantly lower in the low FFM group compared with the healthy FFM group (41.4 ± 7.7 kg vs. 47.4 ± 7.5 kg. but groups did not differ significantly by age or BMI.

While a sample size calculation was not feasible due to a paucity of research exploring appetite responses to such a high intake of protein, this convenience sample was deemed appropriate to allow for the stratifying of the sample for BMI, gender and FFM. The study was sufficiently powered to detect small-medium effects (Cohen’s *f* = 0.3; power = 0.95; α = 0.05).

### 2.2. Experimental Procedure

In a single-blind, within-subject study design, participants completed two experimental conditions one week apart in a randomized, counterbalanced order: high-protein, low-energy content breakfast drink (HPLE) and a very high-protein composition and fed to energy requirements (VHPER). Prior to the main experimental trials, preliminary anthropometric measures were carried out under standardized conditions. Height was measured to the nearest 0.1 cm using a portable stadiometer (Model 213, SECA, Hamburg, Germany) and body mass, measured to the nearest 0.1 kg, and body composition were measured using a multi frequency segmental body composition analyzer (Model BC-418-MA, Tanita Corporation, Tokyo, Japan). On the morning of each trial, participants arrived following an overnight fast (10 h) and having refrained from alcohol consumption and strenuous exercise for 12 h. Subjective appetite sensations were assessed using visual analogue scales (VAS) immediately prior to breakfast drink consumption (0 min) and at 30, 60, 90, 120, 150 and 180 min post-drink, after which participants were free to leave. Participants recorded their EI for the rest of the day using a diet diary. Participants were instructed to refrain from strenuous physical activity for the remainder of the day.

### 2.3. Breakfast Drinks

The breakfast drinks were prepared using vanilla flavored protein powder (XXL Nutrition, Whey Delicious, Helmond, the Netherlands) and semi-skimmed milk. The energy content of the VHPER drink equated to 25% of estimated total energy requirements (TER; 28,29). TER was calculated as estimated basal metabolic rate (BMR), calculated using the Schofield equation [32], multiplied by 1.4 to represent a low level of physical activity. VHPER drink portions were calculated as:

Portion = Breakfast ER (kcal)/285 (kcal per portion)

The semi-skimmed milk and protein powder content of the VHPER drinks were calculated according to the manufacturers recommended serving:

Semi-skimmed milk (mL) = 250 mL × portion

Protein powder (g) = 40 g × portion

The energy and protein content of the HPLE drinks was half that of the VHPER drinks. Water was added to the HPLE drinks to ensure that the volume was identical to the VHPER drinks. Such a low energy content made the HPLE drink representative of the typical low energy breakfast intake of hospitalized older adults at high risk of malnutrition [20]. The energy and macronutrient contents of the HPLE and VHPER drinks are shown in Table 2.

The drinks were weighed to the nearest gram and placed into neutral sealed cups with a straw. Participants were required to consume at least 80% of each drink and failure to do so would result in their withdrawal from the study.

### 2.4. Subjective Appetite Sensations

Appetite perceptions (hunger, fullness, desire to eat and prospective food consumption (PFC)) were measured using previously validated 100 mm VAS [33]. Participants indicated their subjective feelings of appetite by marking a vertical line on the VAS. A composite appetite score was calculated at each time of measurement using the following formula below. Higher composite appetite scores relate to elevated feelings of appetite [34].

[Hunger + (100 − fullness) + desire to eat + prospective consumption]/4(1)

### 2.5. Diet Diaries

Free-living EI was recorded for the reminder of each experimental day using a weighed diet diary. Participants recorded details of all foods and drinks consumed or leftover (g) using calibrated kitchen scales (Disc Electronic Kitchen Scale 1036, Salter Housewares, UK), which were provided. Nutritional analyses were conducted using NetWISP dietary analysis software (version 3.0 for Windows, Tinuviel Software, Anglesey, UK).

### 2.6. Statistical Analysis

Baseline differences in subjective appetite were assessed by a paired-samples *t*-test. Differences in appetite responses between HPLE and VHPER were assessed using a 2 × 7 factorial repeated-measures analysis of variance (ANOVA). Significant interaction and main effects were further explored by pairwise comparisons, using the Bonferroni correction. Area-under-the-curve (AUC) was calculated for appetite profile during the trial period using the trapezoid method, with differences in AUC assessed by paired-samples *t*-test. Differences in free-living energy and protein intake, and in 24 h energy and protein intake (breakfast drink intake + free-living intake) were also assessed using paired-samples *t*-test.

To assess the effect of gender, BMI and fat-free mass (FFM), each was included as a dichotomized between-subject factor in mixed-design ANOVA. For subjective appetite, these analyses were conducted with AUC scores, allowing for a 2 × 2 mixed-design ANOVA (thus removing the second within-subject factor of time and reducing degrees of freedom). In the presence of a significant group main effect for either gender, BMI or FFM, the other two factors were included as a covariate in a mixed-design ANCOVA.

Data are presented as means ± SD in text and as means ± SEM in figures. Throughout, statistical significance was accepted at the level of *p* < 0.05. Where relevant, for *t*-tests and pairwise comparisons, effect size was calculated as Cohen’s d (*d*), with 95% confidence intervals expressed. An effect size of 0.2 or greater was considered small, 0.5 or greater considered medium and 0.8 or greater considered large [35]. For ANOVA, effect size was calculated as partial eta squared (η^2^*_p_*). Data were analyzed using Statistical Package for Social Science (SPSS, Chicago, IL, USA).

## 3. Results

### 3.1. Subjective Appetite

**Figure 1:**
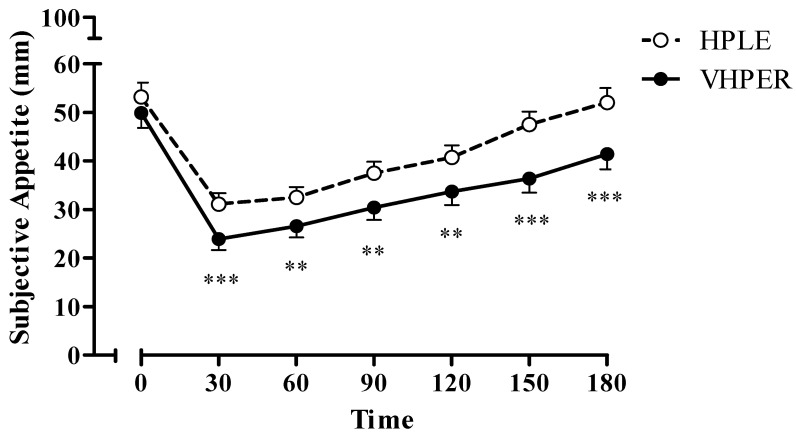
Subjective appetite responses (mean ± SEM) for HPLE (dashed line, clear circles) and VHPER (solid line, black circles). ** *p* < 0.01; *** *p* < 0.001.

Appetite responses for HPLE and VHPER are shown in Figure 1. There were no differences in subjective appetite between the two conditions at baseline. A significant condition x time interaction was observed (F(6, 282) = 2.821, *p* = 0.039, η^2^*_p_* = 0.057). Post hoc pairwise comparisons revealed that subjective appetite was lower in VHPER than HPLE at 30 min and remained lower for rest of the trial (*p* < 0.001 at 30, 150 and 180 min; *p* < 0.01 at 60, 90 and 120 min). Consequently, there was also a condition main effect (mean difference = 7.49 ± 1.64, F(1, 46) = 20.75, *p* < 0.001, η^2^*_p_* = 0.306), and AUC for the appetite profile across the trial period was significantly lower in VHPER, compared with HPLE (32.7 ± 15.3 mm·min^−1^ vs. 40.3 ± 14.1 mm·min^−1^, t(47) = 4.582, *p* < 0.001, *d* = 0.660).

#### Effects of Gender, BMI and FFM

When gender was added as a between-subject factor, there was neither a significant condition x gender interaction (F(1, 46) = 0.484, *p* = 0.490, η^2^*_p_* = 0.010), nor a significant gender main effect (F(1, 46) = 1.161, *p* = 0.287, η^2^*_p_* = 0.05).

When BMI was added as a between-subject factor, there was neither a significant condition x BMI interaction (F(1, 46) = 0.128, *p* = 0.722, η^2^*_p_* = 0.003), nor a significant BMI main effect (F(1, 46) = 0.013, *p* = 0.908, η^2^*_p_* < 0.001).

When FFM was added as a between-subject factor, there was neither a significant condition x FFM interaction (F(1, 41) = 2.498, *p* = 0.122, η^2^*_p_* = 0.057), nor a significant FFM main effect (F(1, 41) = 0.159, *p* = 0.692, η^2^*_p_* = 0.04).

### 3.2. Energy and Protein Intakes

**Figure 2:**
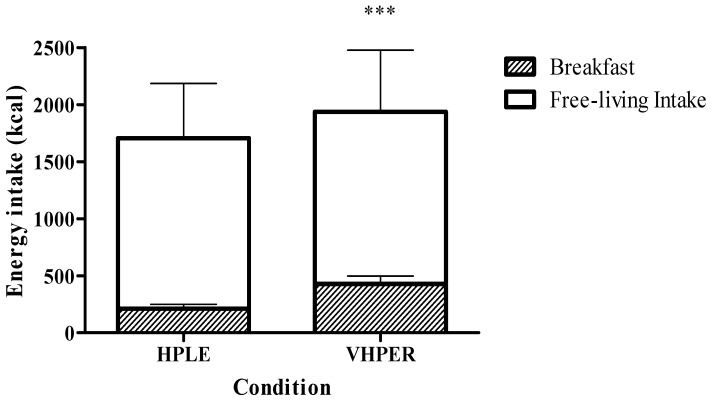
Twenty-four hour (total bar height), breakfast (shaded component of bar) and free-living (clear component of bar) EI (mean ± SEM) for HPLE and VHPER. *** *p* < 0.001.

Energy intake can be seen in Figure 2. Free-living EI did not differ between HPLE and VHPER (1494 ± 479 kcal vs. 1509 ± 538 kcal; t(47) = 0.237, *p* = 0.814). However, when accounting for the breakfast drink, 24 h EI was significantly greater in VHPER condition (1937 ± 568 kcal vs. 1705 ± 490 kcal; t(47) = 3.622, *p* = 0.001, *d* = 0.523).

**Figure 3:**
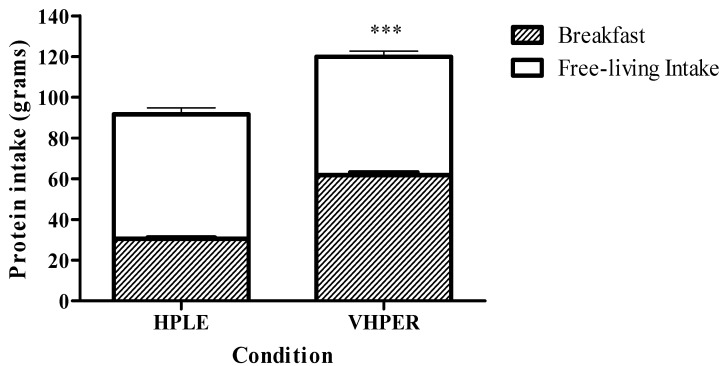
Twenty-four hour (total bar height), breakfast (shaded component of bar) and free-living (clear component of bar) protein intake (mean ± SEM) for HPLE and VHPER. *** *p* < 0.001.

Similarly, free-living protein intake did not differ between HPLE and VHPER (58.1 ± 19.2 g vs. 61.2 ± 22.2 g; t(47) = 1.040, *p* = 0.304). However, when accounting for the breakfast drink, 24 h protein intake was significantly greater in VHPER (123.0 ± 26.0 g vs. 88.6 ± 20.9 g; t(47) = 11.575, *p* < 0.001, *d* = 1.669). Protein intake can be seen in Figure 3.

#### Effects of Gender, BMI and FFM

When adding gender as a between-subject factor, there was no condition x gender interaction for free-living EI, 24 h EI, free-living protein intake or 24 h protein intake (all *p* > 0.1). However, there was a gender main effect for each measure; 24 h EI was greater for men than women (2170 ± 383 kcal vs. 1571 ± 383 kcal; F(1, 46) = 28.552, *p* < 0.001, η^2^*_p_* = 0.383), and men also consumed more protein than women over the 24 h period (119.7 ± 17.7 g vs. 95.8 ± 17.7 g; F(1, 46) = 21.402, *p* < 0.001, η^2^*_p_* = 0.318). However, when adding FFM into an ANCOVA model as a covariate, the only significant gender main effect to remain was 24 h EI (F(1, 44) = 4.203, *p* = 0.047, η^2^*_p_* = 0.095).

Neither free-living nor 24 h EI or protein intake differed by either BMI or FFM (all *p* > 0.05).

## 4. Discussion

The aim of this study was to determine appetite and 24 h food intake responses to manipulating energy and protein intake at breakfast in free-living older adults. Doubling what is generally considered as a “high” protein intake and ensuring an adequate energy content at breakfast resulted in a greater appetite suppression, but did not affect food intake later in the day. Neither free-living energy nor protein intakes across the remainder of the trial day differed between the HPLE or VHPER conditions. Consequently, energy and protein intakes across the 24 h day were significantly greater in VHPER. This suggests there was no compensatory reduction in later-day intake as a result of increased energy and protein intake at breakfast, and that manipulating breakfast to promote high protein consumption and adequate EI can translate into improved protein and energy intake across the day. Our observation supports the findings of Allen et al. [36], who showed that providing high-protein (~14 g) oral nutritional supplements one hour before meals in nursing home-residing and hospitalized dementia patients did not reduce EI at the subsequent meals. In the present study, this was also observed in community-dwelling older adults consuming a much greater amount of protein (~30 g and ~60 g).

The acute appetite responses to the breakfast drinks were independent of gender, BMI or FFM. This is perhaps somewhat unexpected, given that chronic appetite and food intake has been shown to be associated with fat free mass [37,38,39]; one may have expected those with low FFM to have experienced a greater appetite suppression and consequent compromised food intake as a result of the satiating very high protein intake. It should be noted that the energy and protein content of the breakfast drinks were standardized relative to an estimate of participants’ energy requirements (25% of TER), which is determined in part by body size. This may have negated some of the variance in appetite response due to BMI or FFM; however standardizing intake in relative term, as a percentage of energy requirements, rather than in absolute intake terms, is the preferred, valid approach to assessing the primary outcomes of this study (i.e., subjective appetite and energy intake). Other studies which have standardized dietary intake in relative terms (as percentage of energy requirements or habitual intake) prior to assessments of appetite have still observed in relationships between subjective appetite and/or energy intake and FFM [39,40]. The absence of such an observation indicates that breakfast interventions for promoting protein and energy ingestion are likely to be efficacious even in older adults who may already be experiencing the “anorexia of ageing” and energy-protein malnutrition.

The feeding response to different protein and energy intake at breakfast was also observed independent of gender. While it has been evidenced that women typically are more satiated after the same relative intake than men [41,42], young men were more responsive than young women to large loads (30 g and 70 g) of whey protein with regard to hunger suppression and subsequent compensatory decreases in EI [43]. In the study of Davy et al. [44], a pre-lunch yoghurt preload resulted in a greater compensatory decrease in lunch intake in young men, compared with young women, but appetite responses and 24 h compensatory intake did not differ between genders. It did appear that, regardless of breakfast, men ate more than women. However, this 24 h difference in energy and protein intake was primarily driven by the greater breakfast intake (which was calculated based on estimated BMR, which is determined, in part, by body mass); indeed, when controlling for FFM, this gender difference disappeared. As such, it would appear that appetite responses to differing protein and energy intake at breakfast did not differ between older men and women.

The practical implications of these findings for feeding strategies are worth considering. The protein was consumed as whey protein and in liquid form. The supplementation of whey protein, in liquid form, to a typical whole-grain based breakfast meal is a common practice for increasing breakfast protein intake. Whey was the preferred source of protein for optimal stimulation of muscle protein synthesis [45]. While previous research has shown a greater satiety effect of whey supplementation compared with other protein sources [46] such as egg albumin [47] soy [47,48] and casein [48,49], other studies have observed similar appetite responses to protein consumed from a variety of sources, especially when consumed with or in whole foods, as part of a mixed meal [50,51].

It has previously been shown that solid and semi-solid foods are more satiating than energy-matched liquid consumption [52], with increased mastication [53] and greater oro-sensory exposure time [54] postulated as underpinning mechanisms. Leidy and colleagues [55] observed greater appetite suppression in adolescents after a high-protein solid breakfast, compared with a matched high-protein breakfast drink. Participants reported lower postprandial hunger ratings and consumed approximately 115 kcal fewer at an *ad libitum* lunch meal following the solid breakfast versus the breakfast drink. Such greater appetite responses to solid intakes have also been observed in older adults, with a solid meal-replacement product eliciting lower perceptions of hunger and a lower concentration of the potent orexigenic hormone ghrelin, compared with a liquid meal-replacement product [56]. As such, it was deemed most appropriate to deliver the very high protein load of the present study in a potentially less satiating liquid form. The effect of the structure of protein ingestion was not examined, and hence it cannot be determined if similar appetite responses would be observed with very high protein solid food ingestion.

Our findings do partly support the recommendation of 30 g of protein as an achievable target at breakfast for older adults to optimally stimulate muscle protein synthesis [7,57], without considerable impact on appetite. However, alternative, very high-dose protein feeding strategies may provide greater benefits with regards protein intake, without compromising whole-day feeding. The very high protein breakfast intake adopted in this study is an example of pulse feeding. This feeding pattern has previously been implemented as a strategy to ensure energy and protein requirements are met, reducing sarcopenia risk in older adults. In a six-week randomized trial [58,59], pulse feeding was tested in 66 older malnourished or at-risk patients in an inpatient rehabilitation unit. In the control group, daily dietary protein was spread over the four meals. In the pulse diet group, 72% of dietary protein (1.31 g·kg^−1^·day^−1^ on average) was consumed in one meal at noon. Pulse feeding was significantly more efficacious than spread feeding with regard to enhancing amino acid availability [59] and improving lean mass index and appendicular skeletal muscle mass [58]. Our findings support the use of pulse feeding for older adults, suggesting that such a dietary pattern of protein intake does not compromise subsequent feeding due to increased satiety.

While the present study provides important insight into appetite and food intake responses to breakfast manipulation for healthy, free-living adults, the findings can inform future interventions in hospitalized older adults and those in care home facilities. Beerman et al. [60] demonstrated that a protein and energy-enriched breakfast meal (providing 20 g protein and ~500 kcal) did increase total daily protein and energy intakes in hospitalized older adults, but mean intakes still fell short of 75% of calculated protein and energy requirements. Similarly, a protein enriched brioche addition (providing an additional 12.8 g of protein and 180 kcal) to a breakfast meal in a cohort of nursing home residents increased daily protein and energy intakes, but EI remained low and ~one-in-three still failed to achieve minimum protein requirements of 0.8–1.2 g·kg^−1^·day^−1^. In the present study, the VHPER condition resulted in all participants exceeding 1.2 g·kg^−1^·day^−1^ of protein and all but eight participants met total daily energy requirements for a sedentary day. In the HPER condition, nine participants failed to achieve a protein intake of 1.2 g·kg^−1^·day^−1^ and fifteen failed to meet total daily energy requirements. This suggests that, in healthy, free-living older adults at least, a breakfast intervention focused on a very high protein intake but without excessive energy content can contribute to adequate protein and energy intakes throughout the day. This suggests a greater protein intake at breakfast, above the typically considered “high” breakfast portion, and the adoption of a pulse feeding approach, may be of additional benefit for older adults seeking to achieve daily recommended intakes to avoid energy and protein malnutrition. It is of interest to determine if this is the case in hospitalized older adults and those in care home facilities.

The present study is not without limitations. There was no true control condition, and we cannot discount the effects that differences in the carbohydrate nutrient profile of the two breakfast drinks may have had on the study results. The aim of this acute investigation was to compare high- and very high-protein intakes; the benefits of additional protein intake and protein supplementation for older adults at risk of malnutrition are well-established and accepted [13,61], so comparison with a “normal” or habitual protein intake was not deemed necessary for this study. Nonetheless, to determine the long-term effects of a pulse feeding, very high-protein breakfast strategy, comparisons would need to be made with a control condition of habitual breakfast feeding. While subjective appetite and food intake were measured, there was no measure of appetite-associated hormones. The endocrinological regulation of appetite is of interest, especially as it has been shown that older adults exhibit different hormonal responses to feeding than younger adults [62]. However, this study did not aim to determine mechanistic regulation of appetite in response to the varying high protein loads, focusing on perceptive and behavioral responses for such initial investigation. Further, no laboratory measure of food intake was recorded. While it is acknowledged that such measures do afford strong internal validity, preference was for high ecological validity with free-living measures. It was felt that such a focus was important for this acute study to inform future long-term interventions.

## 5. Conclusions

In conclusion, a pulse feeding approach of a very high-protein breakfast intake resulted in a greater appetite suppression than a low-energy, high-protein breakfast intake in older adults; however, there was no compensatory decrease in food intake later in the day, despite the known satiating effect of protein. Consequently, such a protein pulse feeding at breakfast can result in high daily energy and protein intakes. The long-term effects of adopting such a breakfast strategy in older adults at high risk of energy and protein malnutrition warrant exploration.

## Figures and Tables

**Table 1:** Participant characteristics for the whole sample, and when grouped for gender, BMI and FFM.

	**Sample** (***n* = 48**)	
Gender	20 men, 28 women	
Age (years)	69 ± 3	
BMI (kg·m^−2^)	22.2 ± 2.0	
FFM (kg)	45.5 ± 8.0	
		
	Men (*n* = 20)	Women (*n* = 28)
Age (years)	69 ± 3	69 ± 3
BMI (kg·m^−2^)	22.7 ± 1.8	21.9 ± 2.2
FFM (kg)	53.9 ± 4.4	39.9 ± 3.9 ***

	Low BMI (*n* = 31)	Healthy BMI (*n* = 17)
Gender	12 men, 19 women	8 men, 9 women
Age (years)	70 ± 3	69 ± 3
BMI (kg·m^−2^)	21.1 ± 1.4	24.4 ± 1.0 ***
FFM (kg)	44.8 ± 7.7	47.1 ± 8.9
		
	Low FFM (*n* = 14)	Healthy FFM (*n* = 29)
Gender	6 men, 8 women	11 men, 18 women
Age (years)	70 ± 3	69 ± 3
BMI (kg·m^−2^)	21.2 ± 2.3	22.3 ± 1.6
FFM (kg)	41.2 ± 7.7	47.4 ± 7.5 *

FFM: fat-free mass; * *p* < 0.05; *** *p* < 0.001.

**Table 2:** Mean ± SD energy and macronutrient contents of the HPLE and VHPER drinks.

	HPLE	VHPER
Energy (kcal)	211.2 ± 37.1	428.0 ± 68.9
Protein (g)	30.4 ± 5.3	61.8 ± 9.9
Fat (g)	4.7 ± 0.8	9.5 ± 1.5
Carbohydrates (g)	11.9 ± 2.1	23.9 ± 4.0

## Data Availability

Data described in the manuscript will be made available upon request pending approval from Professor Alexandra Johnstone (Professor Johnstone’s contact details can be found here: https://www.abdn.ac.uk/rowett/research/profiles/alex.johnstone, accessed on 7 December 2021).

## Supplementary Materials

The following supporting information can be downloaded at: https://www.mdpi.com/article/10.3390/geriatrics7010016/s1, Figure S1: CONSORT diagram summarizing participant flow. The number of participants who were recruited, enrolled, allocated to intervention, discontinued, included in the analyses and completed are presented.
