# Supplementary material for: The Acute Effects of Breakfast Drinks with Varying Protein and Energy Contents on Appetite and Free-Living Energy Intake in UK Older Adults
Source: Geriatrics (Basel). 2022 Jan 30;7(1):16. doi: 10.3390/geriatrics7010016 (PMC8871635; doi:10.3390/geriatrics7010016)
Supplement: Supplementary file 1 [file geriatrics-07-00016-s001.zip › geriatrics-1523277-supplementary.pdf]

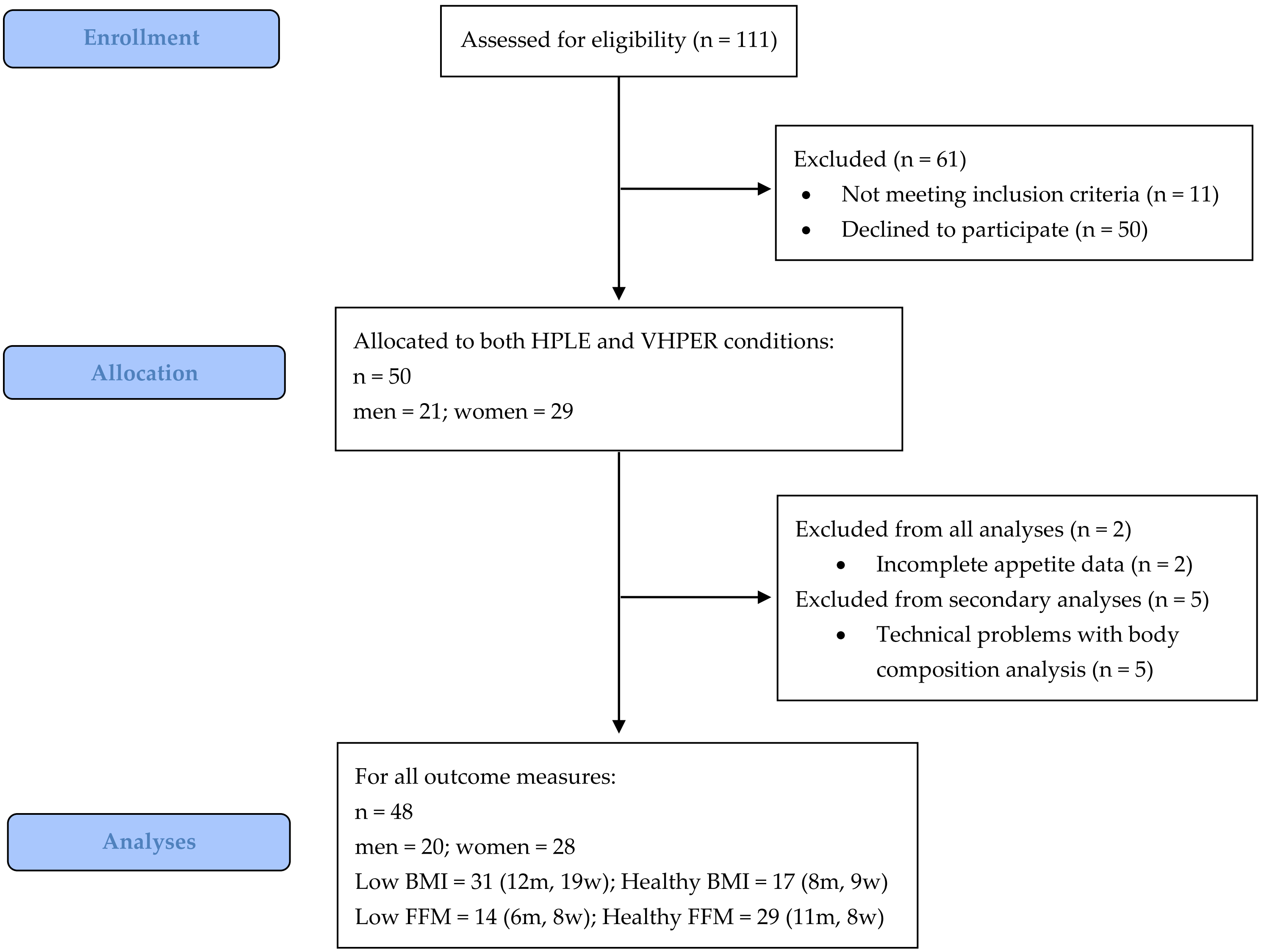

Figure S1. CONSORT diagram summarizing participant flow. The number of participants who were recruited, enrolled, allocated to intervention, discontinued, included in the analyses and completed are presented.
